# Effect of Different Binders on the Electrochemical Performance of Metal Oxide Anode for Lithium-Ion Batteries

**DOI:** 10.1186/s11671-017-2348-6

**Published:** 2017-10-30

**Authors:** Rui Wang, Lili Feng, Wenrong Yang, Yinyin Zhang, Yanli Zhang, Wei Bai, Bo Liu, Wei Zhang, Yongming Chuan, Ziguang Zheng, Hongjin Guan

**Affiliations:** 1 School of Chemistry and Environment, Yunnan Minzu University, Kunming, 650500 China; 2 School of Life and Environmental Sciences, Deakin University, Geelong, VIC 3217 Australia

**Keywords:** Lithium-ion battery, Binder, Anode material, Styrene butadiene rubber, PVDF, Sodium carboxymethyl cellulose, LA133

## Abstract

When testing the electrochemical performance of metal oxide anode for lithium-ion batteries (LIBs), binder played important role on the electrochemical performance. Which binder was more suitable for preparing transition metal oxides anodes of LIBs has not been systematically researched. Herein, five different binders such as polyvinylidene fluoride (PVDF) HSV900, PVDF 301F, PVDF Solvay5130, the mixture of styrene butadiene rubber and sodium carboxymethyl cellulose (SBR+CMC), and polyacrylonitrile (LA133) were studied to make anode electrodes (compared to the full battery). The electrochemical tests show that using SBR+CMC and LA133 binder which use water as solution were significantly better than PVDF. The SBR+CMC binder remarkably improve the bonding capacity, cycle stability, and rate performance of battery anode, and the capacity retention was about 87% after 50th cycle relative to the second cycle. SBR+CMC binder was more suitable for making transition metal oxides anodes of LIBs.

## Background

Lithium ion batteries have become an ideal energy storage equipment and been applied in many portable electronic devices such as mobile phones, audio players, and laptop computers and in aerospace, energy, transportation, and other fields due to the advantages of high specific energy, high working voltage, light quality, long cycle life, small size, and less self-discharge [1–5]. Conventional LIBs use graphite as the anode material which was cheap, abundant, and stable for cycling. However, the further development of graphite LIBs has been hindered due to the low specific capacities (theoretically 372 mAh g^−1^). As a consequence, searching for alternative anode materials was strongly required for the development of advanced LIBs [6, 7]. Recently, 3d transition metal oxides (MO, where M was Fe, Co, Ni, and Cu) were proposed to serve as high theoretic capacity anodes. However, transition metal oxide materials suffered from rapid capacity fade and high initial discharge specific capacity due to the enormous mechanical stress and pulverize during the charge-discharge cycles [8–10]. But during our experiment, we found electrode processing techniques played an important role in improving the cycle stability. In our previous research (2014) [11], octahedral CuO crystals were prepared and used as anode of LIBs, which show high discharge specific capacity and good cycling stability from the 2nd to 50th cycle with the binder of PVDF 301F. But 2 years later, when using PVDF 301F as binder, the same CuO anode showed significantly poor cycle performance that less than 100 mAh g^−1^ after 50 cycles. The detailed reason was not clear, but it was certain that binder played an important role in preparing transition metal oxides anodes and researching the electrochemical performance. In order to enhance the electrochemical performance of lithium-ion batteries, researchers were not only trying to create new electrode materials but also searching for new electrode processing techniques.

The binder was found to be very important, as had also been found by other research groups [12, 13]. Yingjin Wei et al. [14] point out that binder was an important component for battery electrodes whose major function was to act as an effective dispersion agent to connect the electrode species together and then steadily adhere them to the current collectors. They had found when preparing TiO_2_ anode, the electrode using the SBR and CMC as binder had better cycling stability and higher rate performance. M. Mancini’s research group [15] and Shulei Chou’s research group [16] also demonstrated the electrode using CMC as binder had better high-rate capability than the one with PVDF as binder.

**Fig. 1:**
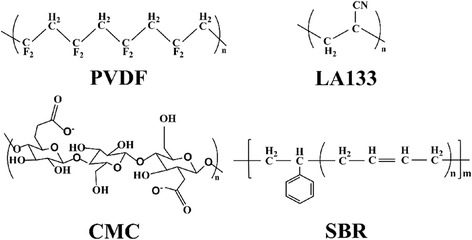
Synopsis of chemical structure of polymers introduce in this work

PVDF was the most commonly used binder for both anode and cathode of LIBs due to the excellent electrochemical and thermal stability and good adhesion between the current collectors and electrode films [17, 18]. Whereas, application prospect of PVDF was limited due to some drawbacks like low flexibility, readily swollen at elevated temperatures, more seriously and also should dissolved in the organic solvent such as *N*-methyl-2-pyrrolidone (NMP), *N*,*N*-dimethylacetamide (DMAc), *N*,*N*-dimethylformamide (DMF). As we know, the most common organic solvent of NMP was expensive, volatile, combustible, toxic, low flexibility, and poor recyclability [19–21]. In the last few years, lots of efforts have been paid attention to seek for alternative water soluble polymers to build up the electrochemical performance. For example, CMC [22, 23], SBR [24], LA133 [25, 26], polyacrylic acid (PAA) [27, 28], polyvinyl alcohol (PVA) [29, 30], polyethylene glycol (PEG has been successfully used in LIBs because it was cheaper, was environmentally friendly, and also has the better solubility) [20], and polyamide imide (PAI) [31] have possibly used water instead of NMP. Among aqueous-based binders, the system based on SBR and CMC was the most studied binder combination and can provide excellent cycling ability and mechanical stability to electrodes when abide the volume expansion during charge-discharge cycling. CMC was a linear polymeric derivative of natural cellulose, the carboxy-methyl (−COO^−^) and hydroxyl (−OH) groups on water soluble contribute to lithium ion exchange in the electrolyte. In addition, SBR as the elastomer show strong binding force, high flexibility, and good heat resistance. So the combination of SBR and CMC can provide high adhesion agent, good cycle performance, strong dispersion medium, and mechanical stability when electrode suffer severe volume expansion during cycling [14, 32]. The chemical structures of the representative binders are shown in Fig. 1. However, which binder was more suitable for preparing transition metal oxides anodes of LIBs have not been systematically researched.

Herein, in this work, to systematically investigate the binding performance between transition metal oxides and the copper foil, five different binders such as PVDF HSV900, PVDF 301F, PVDF Solvay5130, SBR+CMC, and LA133 were used to prepare the anode electrodes (compared to the full battery), and octahedral CuO has been chosen as a representative metal oxide. The electrochemical tests, including constant current charge discharge, cyclic voltammetry, rate performance, and electrochemical impedance spectroscopy were done by statistics. We found SBR+CMC was more suitable for making transition metal oxide anodes of LIBs.

## Experimental

### The Preparation of Anode Electrode

The CuO materials were prepared by a chemical reduction method developed by our group [11]. To fabricate the working electrode, a slurry consisting of CuO materials, carbon black, and binder was usually mixed in certain solvent. When using PVDF as the binder to fabricate the working electrode, a slurry consisting of 60 wt% CuO materials, 10 wt% acetylene black, and 30 wt% PVDF dissolved in NMP was casted on a copper foil, dried at 80 °C for 5 h. When using SBR+CMC as the binder (the CMC was purchased from Hefei Ke Jing materials technology co. LTD., and the viscosity of CMC in 1% aqueous solution was more than 1900 mPa s), a typical formula was that the slurry consisting of 80 wt% CuO materials, 10 wt% acetylene black, 5 wt% SBR, and 5 wt% CMC dissolved in water and was casted on a copper foil, dried at 50 °C for 4 h. When using LA133 (purchased from Chengdu Indigo Power Sources Co., Ltd. China**)** as the binder, a typical formula was that the slurry consisting of 80 wt% CuO materials, 10 wt% acetylene black, and 10 wt% LA133 dissolved in water and was casted on a copper foil, dried at 50 °C for 4 h. Consider that the weight ratio of active materials, carbon black and binder were varied from the choice of different binders.

### Cell Assembly and Electrochemical Studies

The electrochemical measurements were performed with metallic lithium as reference and counter electrode using CR2025 coin cells in an argon-filled glove box with H_2_O and O_2_ concentrations below 1 ppm. The working and counter electrode was separated by Celgard 2320 membrane. The electrolyte was a 1 M solution of LiPF_6_ in ethylene carbonate (EC)-1,2-dimethyl carbonate (DMC) with the ratio of volume 1:1. Galvanostatic charge-discharge was measured on a LAND (CT2001A, China) battery tester. CV and EIS were performed on an electrochemical workstation (CHI604D, Chenhua). The voltage was from 0.01 V to 3.00 V (vs. Li/Li^+^), the current density was 0.2 C, the frequency was ranged from 0.01 to 100 kHz with an AC voltage.

## Results and Discussion

### Galvanostatic Cycling Performance

#### PVDF Binder

**Fig. 2:**
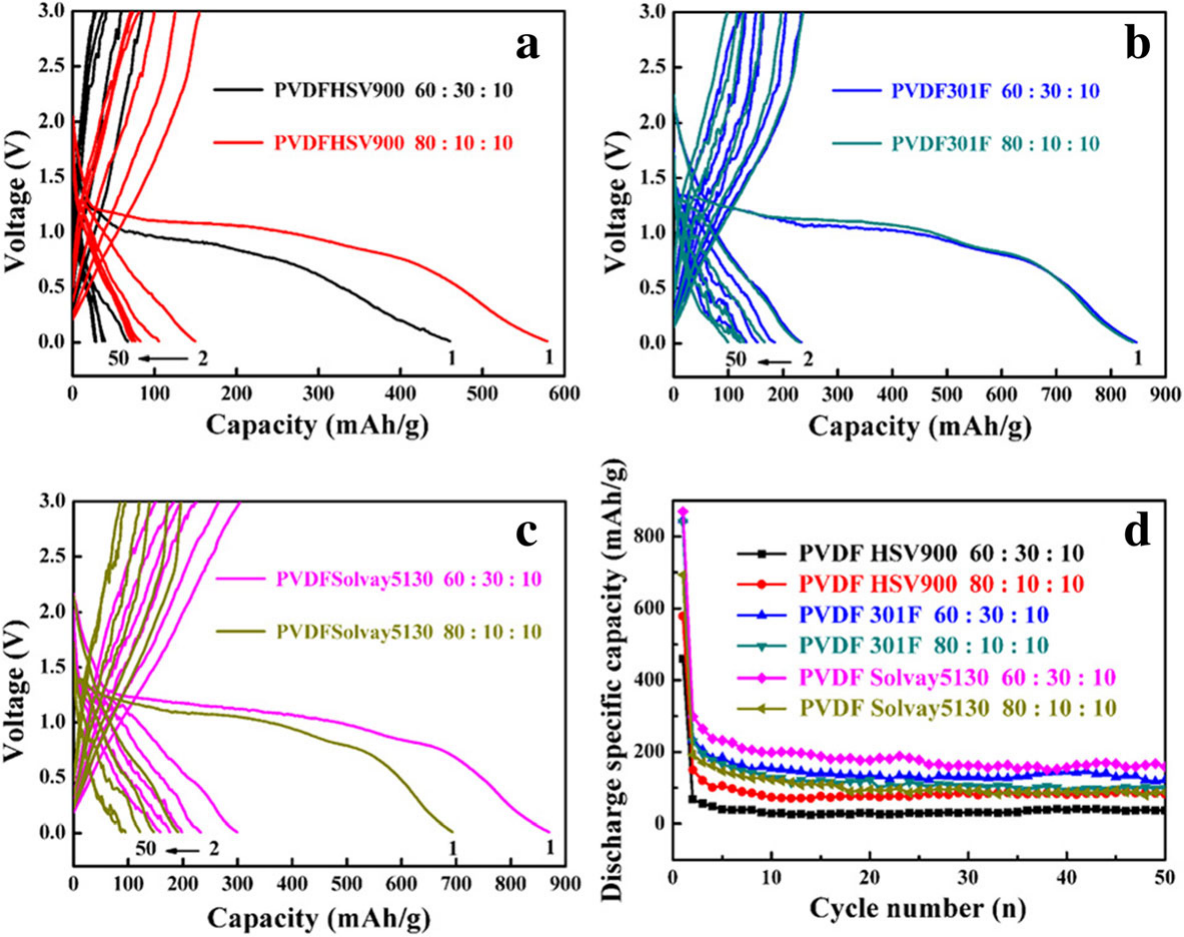
Charge-discharge curves of CuO using different PVDF binders (**a**–**c**) and the cycling performance (**d**). **a** PVDFHSV900, **b** PVDF301F, and **c** PVDFSolvay5130 binder at 0.2 C

The galvanostatic charge-discharge curves of CuO anodes manufactured with PVDF binders (a: HSV900, b: 301F, c: Solvay5130) at 0.2 C rate in the voltage range of 0.01–3.00 V (vs. Li/Li^+^) are shown in Fig. 2. For clarity, the 1st, 2nd, 5th, 10th, 20th, and 50th cycles were the only shown. These results were very different from the previous one [11]. As shown in Fig. 2b, the discharge capacity of CuO anode with PVDF 301F binder in the second cycle was about 250 mAh g^−1^; in addition, the cycling stability was poor and the discharge capacity reduced to less than 100 mAh g^−1^ after 50 cycles. As we know, PVDF was the homopolymer material with high dielectric constant and also has high viscosity and bonding capacity in NMP solvent. The properties of PVDF were different depending on molecular weight. The PVDF of low molecular weight was easy to dissolve, but the performance of battery using PVDF binder was unstable. Most of PVDF molecules can only swelled and not dissolved completely if the molecular weight of PVDF was high (more than 1.2 million), so that the performance of materials cannot be fully played out. Therefore, we bought two new PVDF HSV900 and PVDF Solvay5130 to fabricate CuO anodes. The PVDF of three different molecular weights in the experiment were PVDF HSV900 (about 3 million), PVDF Solvay5130 (1~1.2 million), and PVDF 301F (0.25~1 million) respectively. It can be found that PVDF Solvay5130 and PVDF 301F with smaller molecular weight had the best performance at the slurry ratio of 6:3:1; nevertheless, PVDF HSV900 with larger molecular weight was 8:1:1. It was confirmed that the magnitude of PVDF molecular weight could have important influence on the performance of battery. However, the CuO anodes using three kinds of PVDF as binder show very poor cycle performance beyond our expectation. Although using PVDF Solvay5130 as binder, the CuO anodes show the best cycling performance and discharge capacity; it was a pity that the discharge capacity of the optimal condition in the 1st, 5th, and 50th cycles were 869.7, 298.8, and 158.4 mAh g^−1^, respectively; the capacity retention was lower than 30%. Furthermore, the CuO sample had two well-defined plateau regions in our previous research, whereas here no obvious discharge plateau was observed when using PVDF (a: HSV900, b: 301F, c: Solvay5130) as binders.

Commonly, the reasons for capacity fade of lithium-ion battery anode were as follows [33–35]: (1) the pulverization, over-charge, and discharge in electrode materials, (2) the formation of SEI films in the cycle process on the electrode surface, (3) the decomposition of electrolyte solvent during the discharge process, (4) the irreversible side reaction due to lithium ion inability to remove all, and (5) the slurry fall off copper foil follow the charge-discharge cycles. Here, the preparation condition of CuO anode electrode was identical except the PVDF, so the slurry fell off copper foil follow the charge-discharge cycles may work.

#### SBR+CMC Binder

**Fig. 3:**
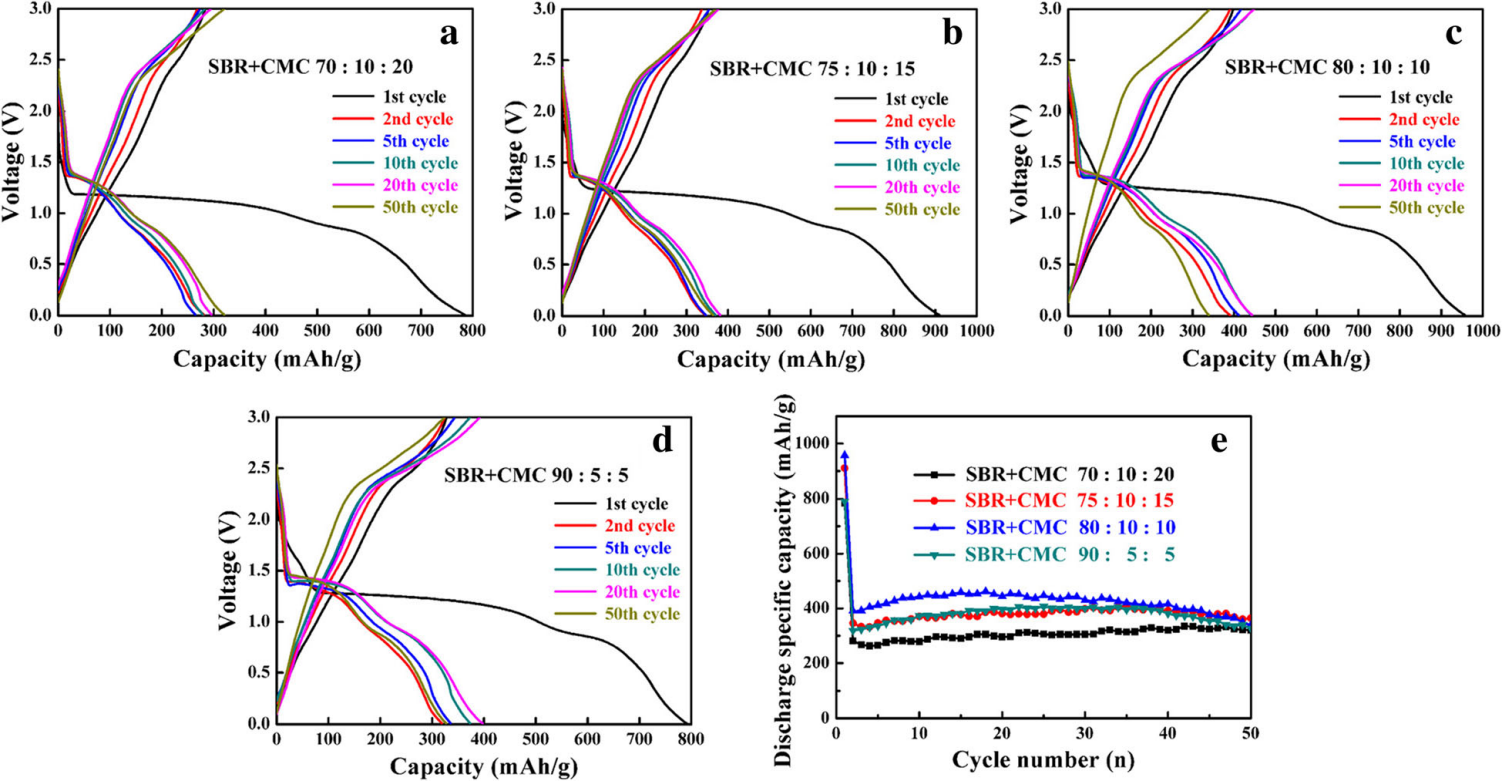
Charge-discharge curves with SBR+CMC binder at different ratios of CuO (**a**–**d**) and the cycling performance (**e**). **a** 70:10:20, **b** 75:10:15, **c** 80:10:10, **d** 90:5:5

Figure 3a–d displays the charge-discharge curves of CuO at 0.2 C and the voltage range of 0.01 to 3.0 V using SBR+CMC binder at the ratio of 70:10:20, 75:10:15, 80:10:10, and 90:5:5, respectively. The viscosity of SBR was too small to be used as single binder so CMC was added to increase viscosity. As shown in Fig. 3, when using SBR+CMC as binder, all the discharge capacities of CuO anodes were much higher than that using PVDF binder. In addition, the cycling stability of CuO anode was improved when using SBR+CMC as binder, especially when the formula was that the slurry consist of 80 wt% CuO materials, 10 wt% acetylene black, and 10 wt% SBR+CMC (as 5 wt% SBR and 5 wt% CMC) as show in Fig. 3e. The CuO anode had the best cycling stability and the highest discharge capacity of 461.3 mAh g^−1^ after 50 cycles and the capacity retention ratio of CuO was about 86.85% that was better than our previous research of 66% [11]. So when making transition metal oxides anodes of LIBs, SBR+CMC binder has bigger cohesion of active materials with the copper foil that was more suitable than PVDF binder. Similar result was reported by Yingjin Wei [6] at 2015; ZnFe_2_O_4_ anode material was prepared via glycine-nitrate combustion method, using SBR+CMC and PVDF as binder in the process of preparing ZnFe_2_O_4_ electrodes. The electrode using SBR+CMC binder exhibit a good capacity retention that the irreversible capacity was 873.8 mAh g^−1^ after 100 cycles, whereas the electrode with PVDF show a serious capacity fade which only retain 461.0 mAh g^−1^ after 15 cycles. Shi-gang Lu et al. [36] have been reported the effect of PVDF and SBR+CMC binder on the electrochemical performance of anode silicon (Si) material. After 30 cycles with the constant current of 200 mAh g^−1^, the reversible capacity of Si electrode using conventional PVDF and elastomeric SBR+CMC as binder was 1093 and 2221 mAh g^−1^, respectively, suggesting that a better capacity retention and an improved cycle performance of Si electrode with SBR+CMC binder. All the data suggest that the cycling stability of battery manufactured with SBR+CMC binder was excellent.

#### LA133 Binder

**Fig. 4:**
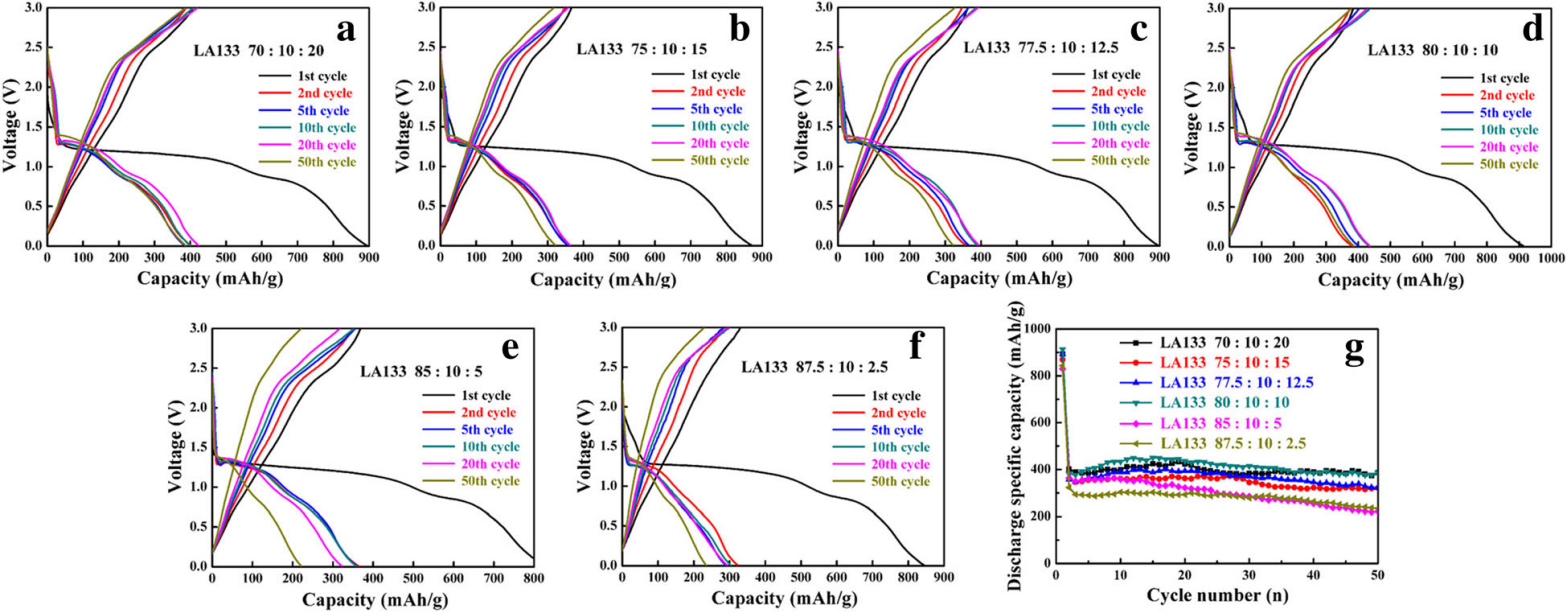
Charge-discharge curves with LA133 binder at different ratios of CuO (**a**–**f**) and the cycling performance (**g**). **a** 70:10:20, **b** 75:10:15, **c** 77.5:10:12.5, **d** 80:10:10, **e** 85:10:5, **f** 87.5:10:2.5

Figure 4a–f present the charge-discharge curves of CuO at 0.2 C and the voltage range from 0.01 to 3.0 V using LA133 for binder at the ratio of 70:10:20, 75:10:15, 77.5:10:12.5, 80:10:10, 85:10:5, and 87.5:10:2.5, respectively. As shown in Fig. 4, when using LA133 as binder, all the cycling stability and discharge capacities of CuO anodes were much higher than using PVDF binder that was much similar to using SBR+CMC binder. When using LA133 as binder, the cycling stability of CuO anode was improved too. In Fig. 4g, the best mixing process of LA133 binder was the slurry ratio of 80:10:10 that exhibit excellent capacity retention ratio about 99% and the discharge capacity was 450.2 mAh g^−1^ after 50 cycles. So LA133 binder was also suitable for making transition metal oxide anodes of LIBs. The main difference between SBR+CMC and LA133 was that SBR+CMC was only applicable for anode electrode and LA133 can be applied to both cathode and anode electrode. The reason of SBR+CMC not can be used in cathode electrode was the unsaturated bond of SBR will be oxidized at high potential, besides the flexibility of the as prepared electrode was also different. When using SBR+CMC as binder, the prepared electrode was more flexible and the round electrode obtained by cutting was relatively smooth and complete. But the prepared electrode using LA133 as binder was brittle and the active material was usually detached from the edge of the electrode at that time cutting to obtain round electrode. Therefore, SBR+CMC was usually selected when preparing anode electrode.

#### Conclusions of Binders

**Fig. 5:**
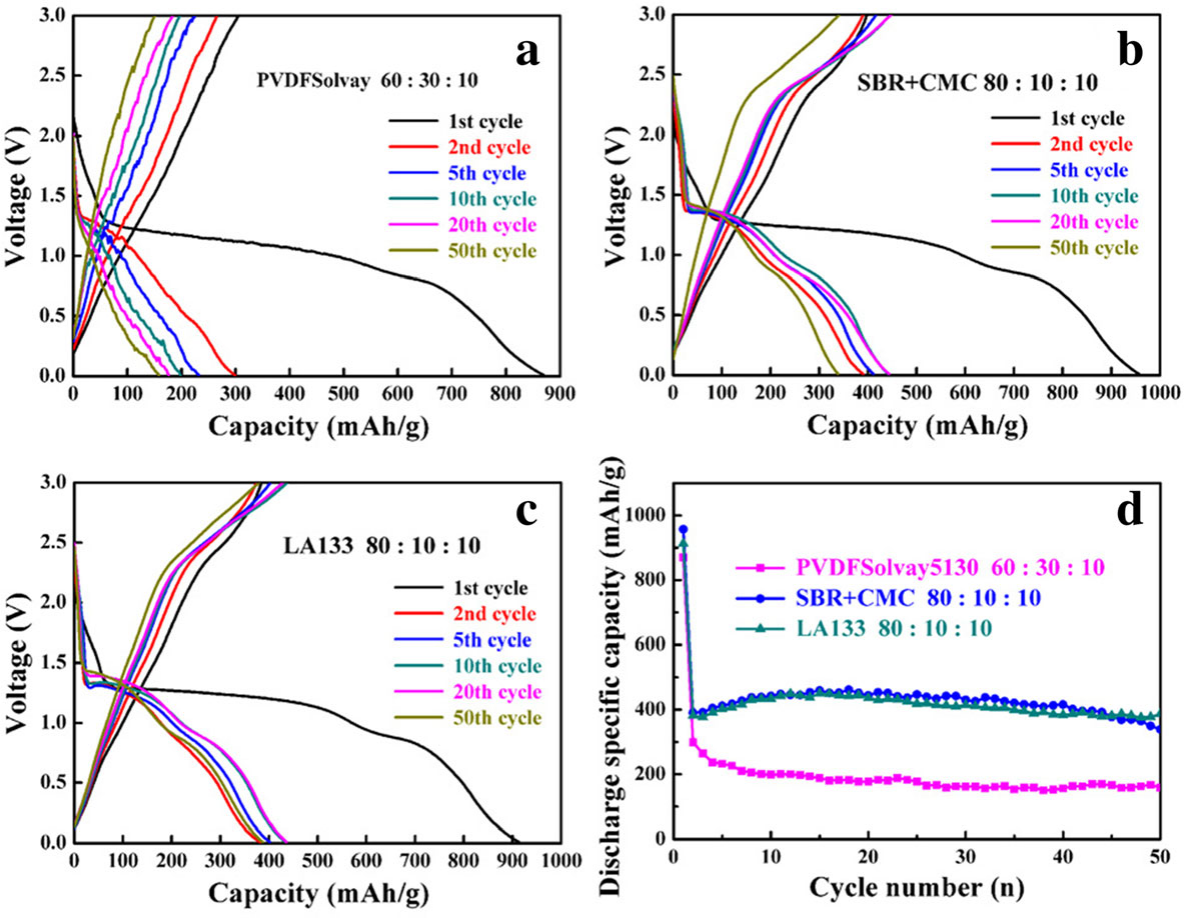
Charge-discharge curves of CuO with different binders (**a**–**c**) and the cycling performance (**d**). **a** PVDF, **b** SBR+CMC, **c** LA133

A deep insight into the cycling performance of the electrodes using three kinds of binder is shown in Fig. 5. It was clear to see that bigger discharge capacities were obtained when using SBR+CMC and LA133 as binder compared to PVDF. The poor electrochemical cycling performance using PVDF as anode binder was also observed by other research group. Zhen Fang et al. [37] synthesized the porous MnCo_2_O_4_ nanorods via a two-step method, through introduction of manganese (Mn) to improve the electrochemical performance of Co_3_O_4_. The influence of binder on the electrochemical performance of MnCo_2_O_4_ anode material have been investigated, which using PVDF as binder show poor performance and fast capacity fade that the discharge capacity was 500 mAh g^−1^ at current density of 0.4 A g^−1^ after 70 cycles. Remarkably, the as-prepared MnCo_2_O_4_ electrode using CMC+SBR exhibit an excellent capacity retention of 1620 mAh g^−1^ at current density of 0.4 A g^−1^ after 700 cycles, even at a high rate of 0.4 A g^−1^~30 A g^−1^ the capacity still up to 533 mAh g^−1^ being cycled at 30 A g^−1^. This indicated that the binder played a significant role in preparing a stable electrode, especially the transition metal oxide materials anode electrode. In conclusion, when made transition metal oxide materials anode electrode for LIBs, PVDF was not a suitable for binder. At this moment, both of SBR+CMC and LA133 were suitable.

### Morphological and Structure Characterization

**Fig. 6:**
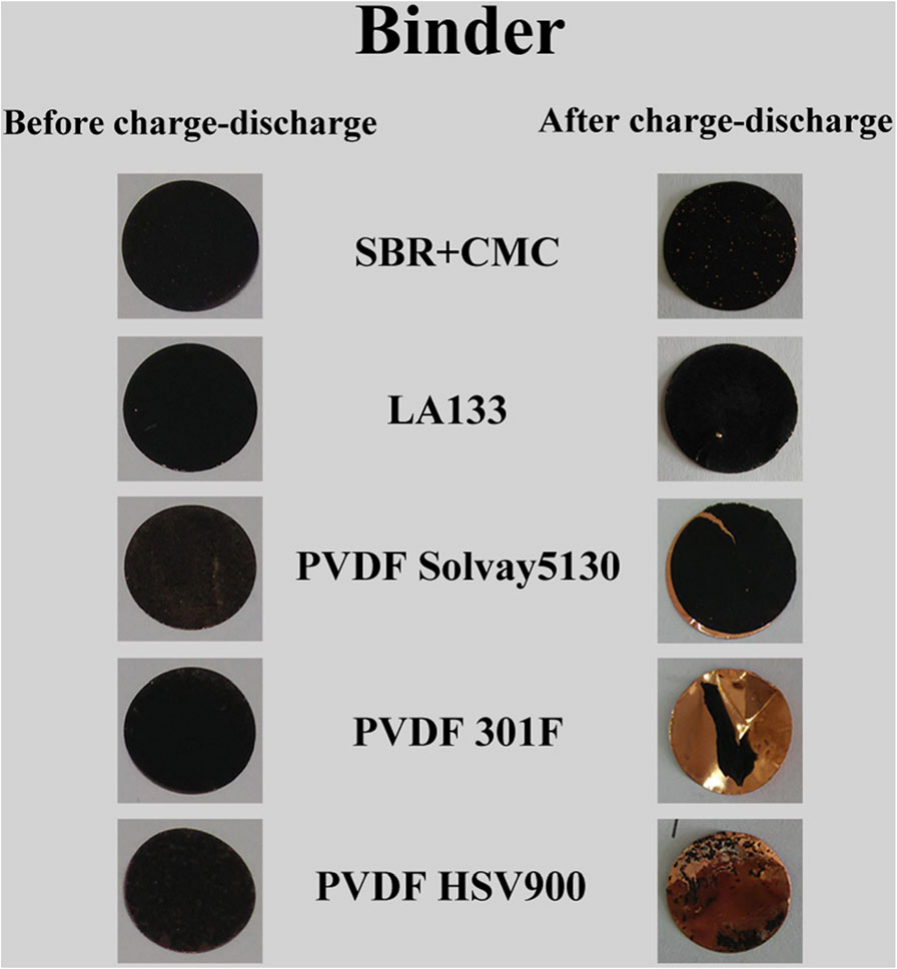
Optical image of CuO electrodes before (left) and after (right) charge-discharge cycles using different binders

In order to have a deep insight into the adhesion of CuO and other active substances on the copper foil, the lithium-ion battery had been opened after galvanostatic charge-discharge test. The optical image of CuO electrodes manufactured with SBR+CMC, LA133, PVDF Solvay5130, PVDF 301F and PVDF HSV900 binders before (left) charge-discharge test and after (right) 50 charge-discharge cycles is shown in Fig. 6; apparently, the electrodes have undergone several changes after several charge-discharge cycles. The electrode films on the latter three electrodes with PVDF binder have obviously fall off from copper foil, and the active substance almost disappeared especially when using PVDF 301F and PVDF HSV900 as binder. By contrast, the electrodes using SBR+CMC and LA133 as binder had not changed too much after 50 charge-discharge cycles, and the adhesion force on copper foil was relatively strong. This related to the adhesion mechanism of PVDF and SBR. When PVDF used as binder, the active material was adhesive to the copper foil in the form of plane bonding, that the adhesion strength was not strong so the entire active material plane was easy to exfoliate from the copper foil. It can be proved by the active material exfoliated from the copper foil integrally as shown in Fig. 6 of using PVDF Solvay5130 binder. When SBR used as binder, the active material bonded to the copper foil in the form of spot bonding, only the active material on this spot can exfoliate from the copper foil when the adhesion strength was not strong. So using SBR+CMC as binder, the cyclic performance of the transition metal oxide materials as the lithium-ion anode should be better in theory.

**Fig. 7:**
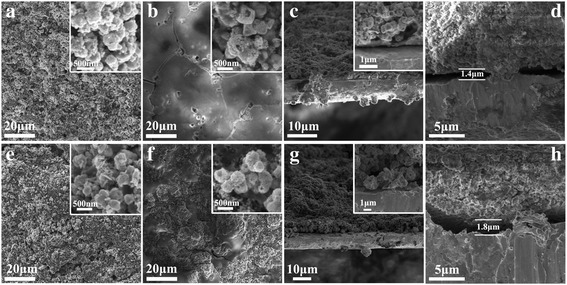
SEM and cross-section SEM image of CuO electrodes using different binders. **a, c** SBR+CMC binder before charge-discharge cycle; **b**, **d** SBR+CMC binder after charge-discharge cycle; **e**, **g** LA133 binder before charge-discharge cycle; **f, h** LA133 binder after charge-discharge cycle

A comparison of CuO electrodes before and after cycle used SBR+CMC (a, b, c, d) and LA133 (e, f, g, h) as binder was analyzed by SEM and is shown in Fig. 7. As the active substance had fallen off from copper foil using PVDF as binder, so the SEM results were not showed. In addition, large magnification figures were equipped in the top right-hand corner of the SEM illustration in order to be analyzed more clearly. The octahedral CuO materials can maintain their octahedral morphology after charge-discharge test. Both the electrode films using SBR+CMC and LA133 binder had attached to the copper foil tightly, especially no gap was found before charge-discharge test as can be seen in Fig. 7c, g. However, a gap was found for both binders between the electrode film and copper foil after charge-discharge test as shown in Fig. 7d, h. When using LA133 binder, the gap between the electrode film and copper foil was about 1.8 μm that much bigger than SBR+CMC binder of 1.4 μm. The gap maybe caused by the immersion in electrolyte and repeated charge and discharge cycles that proved after a long period of cycles, electrode material has the possibility of falling off from the copper foil, but it was still much better than PVDF binder. Therefore, the binder indeed played a very important role in preparation and test of metal oxide anode of LIBs. The outstanding adhesion strength of the mixture of SBR+CMC perhaps can attribute to the three dimensional network by the formation of SBR+CMC. When using SBR+CMC as binder, a stronger polymer chain formed and wrapped around the CuO active material and carbon black. Thus, it can prevent the exfoliation of electrode film from the copper foil.

### Rate Performance

**Fig. 8:**
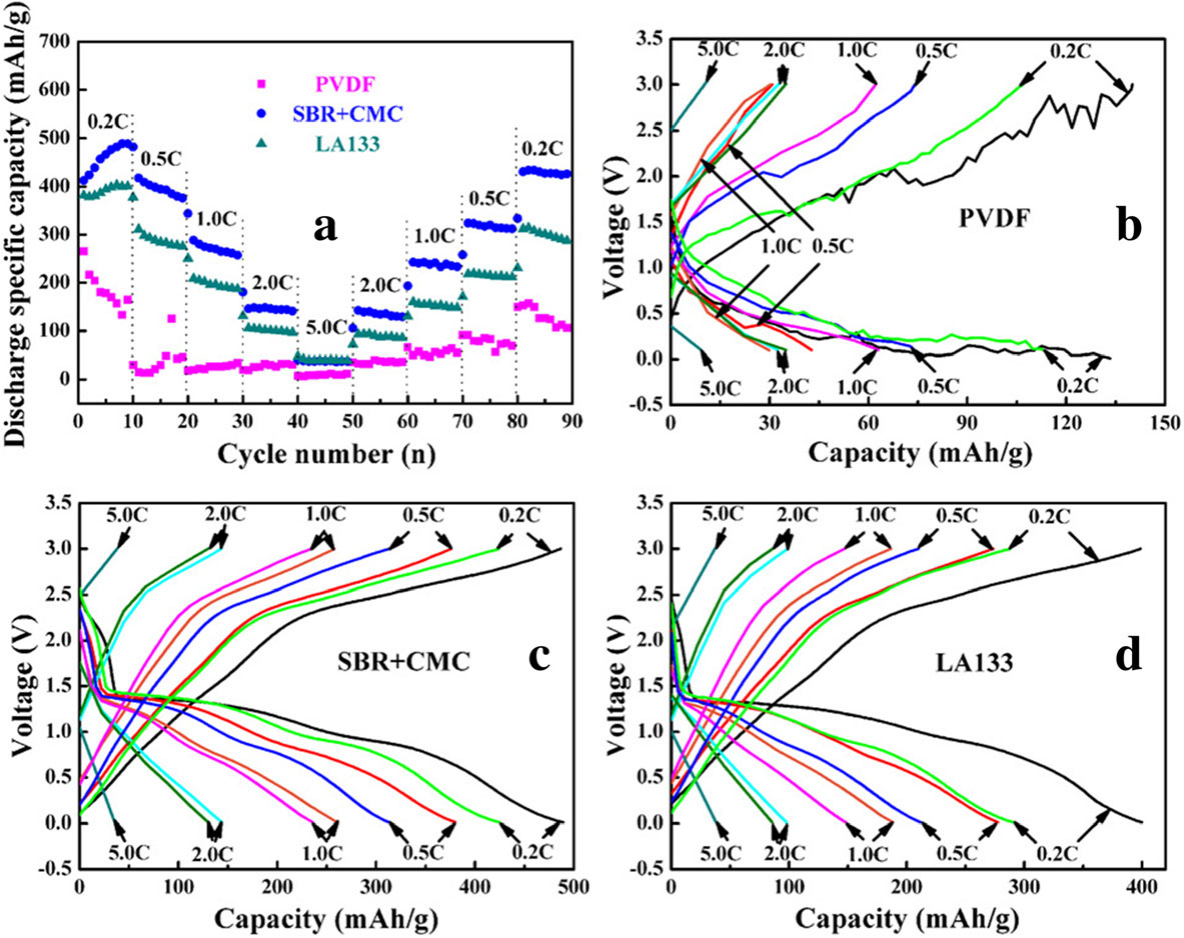
Rate performance (**a**) and the corresponding charge-discharge curves of CuO using different binders. **b** PVDF, **c** SBR+CMC, **d** LA133

The rate performance of CuO electrodes using PVDF, SBR+CMC, and LA133 three kinds of binders under their best condition were shown in Fig. 8. The ratio test process parameter was set to 0.2 C → 0.5 C → 1.0 C → 2.0 C → 5.0 C → 2.0 C → 1.0 C → 0.5 C → 0.2 C to charge and discharge cycle, voltage range of 0.01–3.0 V. Figure 8d compares the cycling performance of three binders at varied current rates; the charge specific capacity of using SBR+CMC binder was much better than PVDF and LA133. The corresponding charge-discharge curves are also shown in Fig. 8a–c, respectively. Almost all the cell capacity had recovered as the current return to the initial low rate of 0.2 C. The recovered capacity of SBR+CMC as binder was 87.0%, which was higher than that of LA133 as binder (71.7%) and PVDF as binder (61.3%). This maybe own to the different dynamics between the three binders.

### Cyclic Voltammetry

**Fig. 9:**
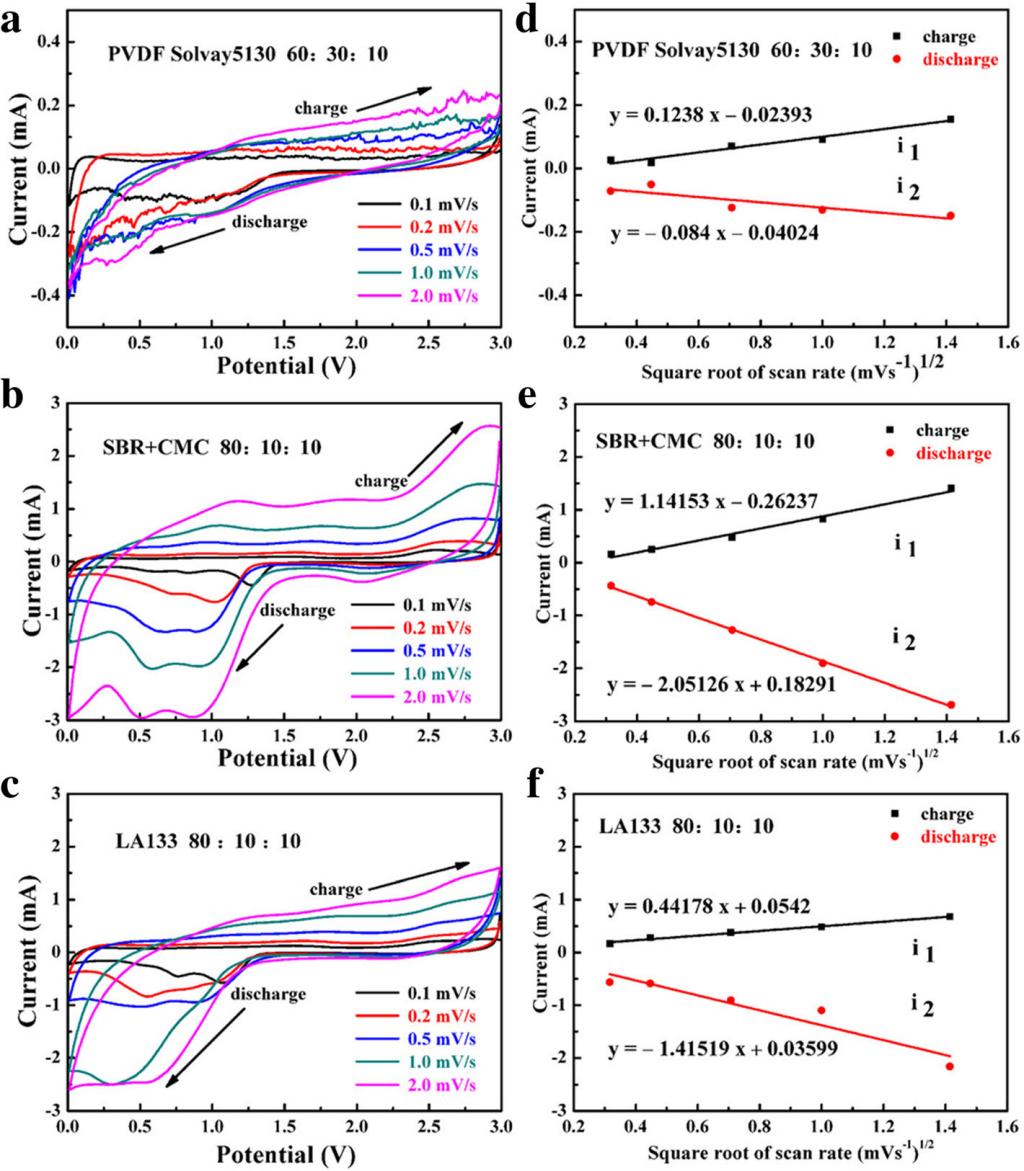
Cyclic voltammograms (left column) of the CuO electrodes using three binders at different scan rates and the relationship between peak current and square root of scan rate (right column). (**a**, **d**) PVDF Solvay5130, (**b**, **e**) SBR+CMC, (**c**, **f**) LAI33

Cyclic voltammograms of CuO electrodes using three kinds of binders under their best condition are shown in the left column of Fig. 9a–c, respectively; asymmetric CV curves indicate that the charge-discharge cycle of battery was not reversible. The scan rate was from 0.1 to 2.0 mV s^−1^ tested after the battery charge-discharge for 2 cycles. The CV graph indicate that there were two obvious reduction peaks appeared at about 0.85 and 1.28 V respectively (especially SBR+CMC binder) when the scan rate was 0.1 mV s^−1^; this indicated the insertion of lithium ion was a two-step reaction and corresponding to the two discharge platforms of discharge curve. The reduction peak located at the potential of 1.28 V was corresponding to the transformation of CuO to Cu_2_O, and the reduction peak located at the potential of 0.85 V was corresponding to the transformation of Cu_2_O to Cu. In addition, a small reduction peak appeared at 2.25 V, which attributed to the creation of SEI with CuO phase [38–40]. In charge process, two of the oxidation peaks cannot be easily distinguished. They merged into an oxidation peaks at 2.54 V, which related to the transformation process of Cu to Cu (I) and Cu (II). In addition, an unobvious broad peak around 1.50 V may correspond to the decomposition of SEI layer. With increasing the scanning speed, the two reducing peaks moved to negative potential and the irreversibility increased. When SBR+CMC used as binder, the irreversibility of the oxidation and reduction peak were the minimum, indicating the lowest electrochemical polarization. When PVDF and LA133 used as binder, the peak shape became less and less clear with increasing scanning speed. Whereas when SBR+CMC used as binder, the oxidation and reduction peak were very obvious even at 2.0 mV s^−1^. The good peak shape in cyclic voltammogram test proved that SBR+CMC binder was better than PVDF and LA133. Furthermore, through the contrast, it can be obtained that the peak current and peak area using SBR+CMC as binder were much bigger than that using PVDF and LA133 as binder.

To further research the electrode kinetics, the lithium diffusion coefficient of CuO electrode using different binders can be calculated from the Randles-Sevcik equation [41].

1 $$ {i}_p=0.4463 nFAC{\left( nFvD/ RT\right)}^{1/2} $$

According to Eq. 1, *i*_p_ was indicative of the peak current (A), *n* was the number of the electrons in the process of transfer, *F* represents the Faraday constant (96,486 C mol^−1^), *A* was the electrode area (cm^2^), *C* stands for the volume concentration (mol cm^−3^), *ν* represents the sweep speed (V s^−1^), *D* on behalf of the diffusion coefficient (cm^2^ s^−1^), *R* was the gas constant (8.314 J K^−1^ mol^−1^), and *T* represents the test temperature (K). When the room temperature was 25 °C, put the *F* and *R* into Eq. (1):

2 $$ {i}_p=268600{n}^{3/2}{AD}^{1/2}{Cv}^{1/2} $$

From the type, it can be found the peak current was in direct ratio with the square root of scan rate and the slope of the straight line corresponding to the 268600n^3/2^AD^1/2^C in the formula.

Figure 9d–f shows the good linear relationship of *i*_p_ and ν^1/2^ for CuO electrodes using PVDF, SBR+CMC, and LA133 for binders, respectively. The diffusion coefficient on the progress of insertion and extraction of Li^+^ in CuO were calculated by the biggest oxidation peak (about 2.54 V at charge process when using SBR+CMC binder) and reductive peak (about 1.28 V at discharge process when using SBR+CMC binder), and the corresponding results based on Eq. (2) are listed in Table 1. It was can be seen from Table 1 that the value of Li^+^ diffusion coefficient in CuO electrode using SBR+CMC binder was much higher than the others both on the charge and discharge cycles. The larger value indicated that the use of SBR+CMC for binder was more beneficial to the intercalation kinetics of lithium ion, which also can explain why using SBR+CMC as binder has better electrochemical performance than PVDF and LA133 binder.

**Table 1:** Diffusion coefficients of Li^+^ of CuO using different binders. D_1_, Diffusion coefficients of Li^+^ in charge (oxidation) process; D_2_, Diffusion coefficients of Li^+^ in discharge (reductive) process

Binder	D_1_/cm^2^ s^−1^	D_2_/cm^2^ s^−1^
PVDF Solvay5130	0.2 × 10^−8^	0.1 × 10^−8^
SBR+CMC	6.6 × 10^−8^	21.5 × 10^−8^
LA133	0.6 × 10^−8^	6.2 × 10^−8^

### Electrochemical Impedance Spectroscopy

**Fig. 10:**
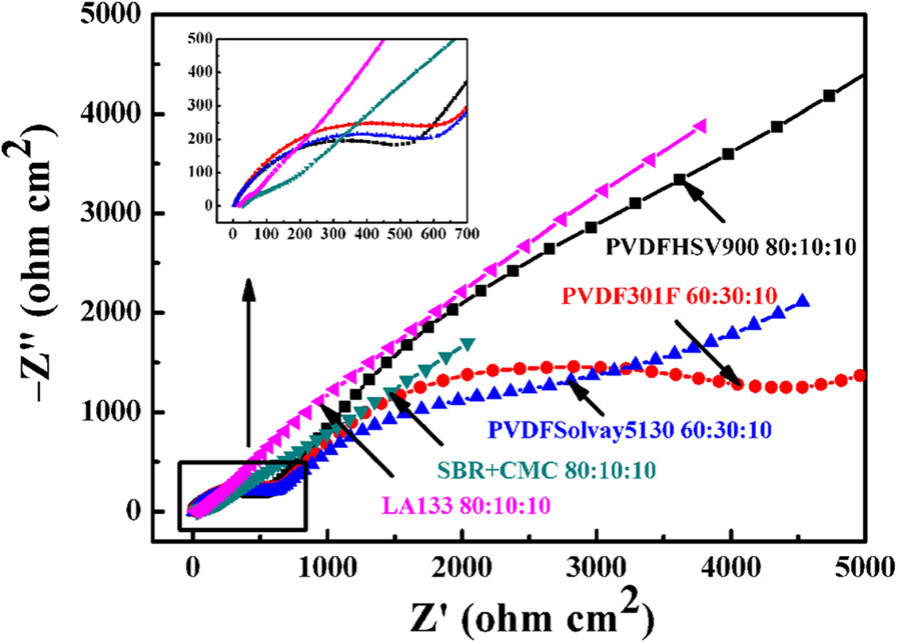
Electrochemical impedance spectra of CuO electrodes using different binders after 50 charge-discharge cycles

In order to study the electrochemical kinetics and conductivity of the CuO electrode material using different binders, EIS measurements were carried out at the open circuit voltage with the frequency ranging from 0.01 to 100 Hz and the AC impedance was 5 mV. Before EIS tests, all cells were constant current charge-discharged for 50 cycles. The Nyquist plots of CuO using different binders are displayed in Fig. 10. Obviously, the EIS spectra was composed of a circle in the high-frequency area and a slash in the low-frequency region. The intercept on the Z′ real axis represented the ohmic resistance (R_s_) that corresponds to the resistance of electrolyte. The semicircle in the high frequency corresponds to the resistance of the SEI film (R_sf_) and the charge transfer resistance (R_ct_). The line stands for the Warburg impedance (W_s_) which is in connection with the Li^+^ diffusion in active materials. It can be observed in Fig. 10 that the resistance of the semicircle with SBR+CMC and LA133 had similar value about 50 Ω cm^2^ which was much smaller than the PVDF. So little resistance indicated faster charge transfer for CuO electrode and also demonstrated that using SBR+CMC as binder was conducive to a rapidly electrochemical reaction and preferable capacity retention of active materials.

Moreover, electric conductivity of CuO electrodes using different binders can also be tested by AVO meter, and the corresponding measurement result is listed in Table 2. The results showed that using SBR+CMC as binder had smallest electrical resistance, which the value of 200 Ω was smaller compared with other binders especially than the PVDFHSV900 (500,000 Ω).

**Table 2:** Conductivity results of CuO electrodes using different binders

Binder	SBR+CMC	LA133	PVDF Solvay5130	PVDF 301F	PVDF HSV900
Resistance (Ω)	200	300	500	800	500,000

## Conclusions

In conclusion, this study has investigated the electrochemical performance of CuO electrodes handle with different binders and also researched the adhesive properties of the organic PVDF binders or aqueous binders of SBR+CMC and LA133 can be varied over the weight ratio of conductive slurry. Test results show that active material was easy to fall off from the current collector if use PVDF for binder. By contrast, SBR+CMC and LA133 displayed the preferable bonding performance. It can be observed that fabricated with SBR+CMC binder, especially when the slurry ratio was 80:10:10, the electrode demonstrated an outstanding electrical conductivity, excellent charge transfer, prominent binding capability, remarkable cycling performance, and good rate performance, and eventually result in the brilliant electrochemical performance. Consequently, this work provided the experimental feasibility and theoretical proof of manufacturing LIBs anode materials using cheap aqueous SBR+CMC binder instead of poisonous solvent like NMP and expensive PVDF. Hence, the battery electrochemical property be promoted, cost be reduced, and environment be protected accordingly.

## References

[1] Xiao W, Gong Y, Wang H, Liu J, Yan C (2016). Organic-inorganic binary nanoparticles-based composite separator for high performance lithium-ion batteries. New J Chem.

[2] Dirican M, Yanilmaz M, Fu K, Yildiz O, Kizil H, Hu Y, Zhang X (2014). Carbon-confined PVA-derived silicon/silica/carbon nanofiber composites as anode for lithium-ion batteries. J Electrochem Soc.

[3] Gao G, Zhang Q, Wang K, Song H, Qiu P, Cui D (2013). Axial compressive α-Fe_2_O_3_ microdisks prepared from CSS template for potential anode materials of lithium ion batteries. Nano Energy.

[4] Sun J, Lv C, Lv F, Chen S, Li D, Guo Z, Han W, Yang D, Guo S (2017). Tuning the shell number of multishelled metal oxide hollow fibers for optimized lithium-ion storage. ACS Nano.

[5] Wei Q, Xiong F, Tan S, Huang L, Lan EH, Dunn B, Mai L (2017). Porous one-dimensional nanomaterials: design, fabrication and applications in electrochemical energy storage. Adv Mater.

[6] Zhang R, Yang X, Zhang D, Qiu H, Fu Q, Na H, Guo Z, Du F, Chen G, Wei Y (2015). Water soluble styrene butadiene rubber and sodium carboxyl methyl cellulose binder for ZnFe_2_O_4_ anode electrodes in lithium ion batteries. J Power Sources.

[7] An Q, Lv F, Liu Q, Han C, Zhao K, Sheng J, Wei Q, Yan M, Mai L (2014). Amorphous vanadium oxide matrixes supporting hierarchical porous Fe_3_O_4_/graphene nanowires as a high-rate lithium storage anode. Nano Lett.

[8] Zhang X, Hu Y, Zhu D, Xie A, Shen Y (2016). A novel porous CuO nanorod/rGO composite as a high stability anode material for lithium-ion batteries. Ceram Int.

[9] Xu L, Sitinamaluwa H, Li H, Qiu J, Wang Y, Yan C, Li H, Yuan S, Zhang S (2016). A low cost and green preparation process of α-Fe_2_O_3_ @gum Arabic electrode for high performance sodium ion battery. J Mater Chem A.

[10] Gao G, Zhang Q, Cheng X, Qiu P, Sun R, Yin T (2015). CNTs in situ attached to α-Fe_2_O_3_ submicron spheres for enhancing lithium storage capacity. ACS Appl Mater Interfaces.

[11] Feng L, Xuan Z, Bai Y, Zhao H, Li L, Chen Y, Yang X, Su C, Guo J, Chen X (2014). Preparation of octahedral CuO micro/nanocrystals and electrochemical performance as anode for lithium-ion battery. J Alloys Compd.

[12] Rey-Raap N, Piedboeuf M-LC, Arenillas A, Menéndez JA, Léonard AF, Job N (2016). Aqueous and organic inks of carbon xerogels as models for studying the role of porosity in lithium-ion battery electrodes. Mater Des.

[13] Pohjalainen E, Sorsa O, Juurikivi J, Kallio T (2016). Water-soluble acrylate binder for graphite electrodes in lithium-ion batteries. Energy Technol.

[14] Yan X, Zhang Y, Zhu K, Gao Y, Zhang D, Chen G, Wang C, Wei Y (2014). Enhanced electrochemical properties of TiO_2_(B) nanoribbons using the styrene butadiene rubber and sodium carboxyl methyl cellulose water binder. J. Power Sources.

[15] Mancini M, Nobili F, Tossici R, Wohlfahrt-Mehrens M, Marassi R (2011). High performance, environmentally friendly and low cost anodes for lithium-ion battery based on TiO_2_ anatase and water soluble binder carboxymethyl cellulose. J Power Sources.

[16] Chou S-L, Wang J-Z, Liu H-K, Dou S-X (2011). Rapid synthesis of Li_4_Ti_5_O_12_ microspheres as anode materials and its binder effect for lithium-ion battery. The J Phys Chem C.

[17] Song J, Zhou M, Yi R, Xu T, Gordin ML, Tang D, Yu Z, Regula M, Wang D (2014). Interpenetrated gel polymer binder for high-performance silicon anodes in lithium-ion batteries. Adv Funct Mater.

[18] Wang N, NuLi Y, Su S, Yang J, Wang J (2017). Effects of binders on the electrochemical performance of rechargeable magnesium batteries. J Power Sources.

[19] Zhong H, Sun M, Li Y, He J, Yang J, Zhang L (2016). The polyacrylic latex: an efficient water-soluble binder for LiNi_1/3_Co_1/3_Mn_1/3_O_2_ cathode in li-ion batteries. J Solid State Electrochem.

[20] Tran B, Oladeji IO, Wang Z, Calderon J, Chai G, Atherton D, Zhai L (2013). Adhesive PEG-based binder for aqueous fabrication of thick Li_4_Ti_5_O_12_ electrode. Electrochim Acta.

[21] Tran B, Oladeji IO, Wang Z, Calderon J, Chai G, Atherton D, Zhai L (2012). Thick LiCoO_2_/nickel foam cathode prepared by an adhesive and water-soluble PEG-based copolymer binder. J Electrochem Soc.

[22] Lux SF, Schappacher F, Balducci A, Passerini S, Winter M (2010). Low cost, environmentally benign binders for lithium-ion batteries. J Electrochem Soc.

[23] Wang Z, Dupre N, Gaillot A-C, Lestriez B, Martin J-F, Daniel L, Patoux S, Guyomard D (2012). CMC as a binder in LiNi_0.4_Mn_1.6_O_4_ 5 V cathodes and their electrochemical performance for Li-ion batteries. Electrochim Acta.

[24] Kvasha A, Urdampilleta I, Id M, Bengoechea M, Blázquez JA, Yate L, Miguel O, Grande H-J (2016). Towards high durable lithium ion batteries with waterborne LiFePO_4_ electrodes. Electrochim Acta.

[25] Wei Y, Tao Y, Kong Z, Liu L, Wang J, Qiao W, Ling L, Long D (2016). Unique electrochemical behavior of heterocyclic selenium sulfur cathode materials in ether based electrolytes for rechargeable lithium batteries. Energy Storage Mater.

[26] Pang C, Ding F, Sun W, Liu J, Hao M, Wang Y, Liu X, Xu Q (2015). A novel dimethyl sulfoxide/1,3-dioxolane based electrolyte for lithium/carbon fluorides batteries with a high discharge voltage plateau. Electrochim Acta.

[27] Zhang Z, Zeng T, Qu C, Lu H, Jia M, Lai Y, Li J (2012). Cycle performance improvement of LiFePO_4_ cathode with polyacrylic acid as binder. Electrochim Acta.

[28] Gangaja B, Chandrasekharan S, Vadukumpully S, Nair SV, Santhanagopalan D (2017). Surface chemical analysis of CuO nanofiber composite electrodes at different stages of lithiation/delithiation. J Power Sources.

[29] Prosini PP, Carewska M, Cento C, Masci A (2014). Poly vinyl acetate used as a binder for the fabrication of a LiFePO_4_-based composite cathode for lithium-ion batteries. Electrochim Acta.

[30] Sreelakshmi KV, Sasi S, Balakrishnan A, Sivakumar N, Nair AS, Nair SV, Subramanian KRV (2014). Hybrid composites of LiMn_2_O_4_–Graphene as rechargeable electrodes in energy storage devices. Energy Technol.

[31] Yang HS, Kim S-H, Kannan AG, Kim SK, Park C, Kim D-W (2016). Performance enhancement of silicon alloy-based anodes using thermally treated poly(amide imide) as a polymer binder for high performance lithium-ion batteries. Langmuir.

[32] Chou W-Y, Jin Y-C, Duh J-G, Lu C-Z, Liao S-C (2015). A facile approach to derive binder protective film on high voltage spinel cathode materials against high temperature degradation. Appl Surf Sci.

[33] Su D, Xie X, Dou S, Wang G (2014). CuO single crystal with exposed {001} facets—a highly efficient material for gas sensing and Li-ion battery applications. Sci Rep.

[34] Cao F, Xia XH, Pan GX, Chen J, Zhang YJ (2015). Construction of carbon nanoflakes shell on CuO nanowires core as enhanced core/shell arrays anode of lithium ion batteries. Electrochim Acta.

[35] Bai Z, Zhang Y, Zhang Y, Guo C, Tang B (2015). A large-scale, green route to synthesize of leaf-like mesoporous CuO as high-performance anode materials for lithium ion batteries. Electrochim Acta.

[36] Li T, Yang J-y, Lu S-g (2012). Effect of modified elastomeric binders on the electrochemical properties of silicon anodes for lithium-ion batteries. Int J Min Met Mater.

[37] Zeng P, Wang X, Ye M, Ma Q, Li J, Wang W, Geng B, Fang Z (2016). Excellent lithium ion storage property of porous MnCo_2_O_4_ nanorods. RSC Adv.

[38] Yang W, Wang J, Ma W, Dong C, Cheng G, Zhang Z (2016). Free-standing CuO nanoflake arrays coated cu foam for advanced lithium ion battery anodes. J Power Sources.

[39] Subalakshmi P, Sivashanmugam A (2017). CuO nano hexagons, an efficient energy storage material for Li- ion battery application. J Alloys Compd.

[40] Zhang R, Liu J, Guo H, Tong X (2015). Synthesis of CuO nanowire arrays as high-performance electrode for lithium ion batteries. Mater Lett.

[41] Kim A-Y, Kim MK, Cho K, Woo J-Y, Lee Y, Han S-H, Byun D, Choi W, Lee JK (2016). One-step catalytic synthesis of CuO/Cu_2_O in a graphitized porous C matrix derived from the cu-based metal−organic framework for Li- and Na-ion batteries. ACS Appl Mater Interfaces.

